# Ruminal impaction due to Ficus esquiroliana Levl. in Boer goats

**DOI:** 10.1186/2193-1801-2-608

**Published:** 2013-11-14

**Authors:** Shao-Lun Zhai, Xiao-Hui Wen, Man-Lin Luo, Dian-Hong Lv, Wen-Kang Wei

**Affiliations:** Institute of Animal Health, Guangdong Academy of Agricultural Sciences, Baishigang Street, Wushan Road, Tianhe District Guangzhou, 510640 China; College of Veterinary Medicine, South China Agricultural University, Guangzhou, 510642 China

**Keywords:** Ruminal impaction, *Ficus esquiroliana* Levl, Boer goat

## Abstract

Ruminal impaction is considered an important internal disease in ruminants, such as dairy cows, sheep, and goats. It has been reported that its occurrence is associated with many different causes. This study describes a novel case of ruminal impaction caused by a plant, *Ficus esquiroliana* Levl., in Boer goats. This case suggests that *Ficus esquiroliana* should be taken into consideration when providing food for ruminants.

## Introduction

The Boer goat, which was introduced from Germany in 1998, is currently considered as one of the most important goat breeds in China. Due to its good productive performance and strong resistance to infectious disease, the Boer goat has found an important economic and ecological niche in agricultural systems throughout the developing countries, including China (Devendra, 2005). However, certain internal diseases often lead to major economic losses for farms. Ruminal impaction is an important internal disease in goats, which often leads to acute death in young and adult goats, and to chronic death in lambs due to the inability to acquire milk from ewes. Herein, a novel case of ruminal impaction in Boer goats due to the plant *Ficus esquiroliana* Levl. is reported.

## Case description

**Figure 1:**
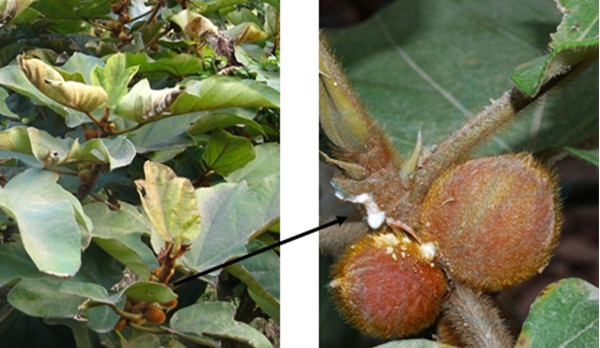
**Branches, leaves, and fruit of** ***Ficus esquiroliana*** **Levl. (from the China Plant Image Library, PlantPhoto.cn).**

On January 20, 2013, a farm owner from Heyuan City, Guangdong Province (250 km from Guangzhou City), reported that there were approximately 200 adult Boer goats, 100 young Boer goats, and 50 lambs on the farm. Typically, these goats are fed once per day via ad libitum grazing on a hillside (approx. 600 hectares). However, over a period of 2 days, breeders did not provide free grazing, but harvested many branches and leaves (10–12 kg) of *Ficus esquiroliana* (Figure 1) to feed these goats in a sheepfold. Following this change in feeding, some goats (approx. 30%) showed some clinical signs of disease, such as listlessness, less rumination, or no rumination. The goats subsequently exhibited ruminal impaction and ruminal tympany, before finally showing increased salivation, difficulty in breathing, no breathing, and/or death. Many lambs had systemic failure and died slowly due to their inability to obtain milk from ewes. Pathological changes showed that the rumen of dead goats had become firm and filled with many viscous contents, including the branches and leaves of *Ficus esquiroliana*. Moreover, almost all the mucosa of the rumen for dead goats was easily peeled off. In addition, there were many dry contents presenting in other stomach compartments including (reticulum, omasum and abomasums) and even in the small intestines.

For those ailing goats with no rumination, aseptic surgery of the left para lumbar fossa was performed with the administration of a local infiltration of lignocain solution. The incision revealed that the rumen was severely impacted with many branches and leaves of *Ficus esquiroliana*, which were removed through a laparotomy incision. 5% sugar-salt-water (2–3 L), vitamin K (20 mL, 1 ml:10 mg), and 5% sodium bicarbonate injection (1 L) were then infused into the body of the goats intravenously to prevent the occurrence of dehydration and acidosis. Generally speaking, the sick goats with complete mucosa of the rumen had a good prognosis. Moreover, the farmers were advised to alter the diet of goats that were still alive, but showing signs of the disease.

## Discussion

In China, *Ficus esquiroliana*, a species of subtropical tree, is mainly distributed in Tibet, Xichuan, Yunan, Guangxi, Guangdong, Hainan, Fujian, and Taiwan. Moreover, it occurs in the northern regions of India, Vietnam, Laos, and Thailand. The roots and bark of *Ficus esquiroliana* are used as a drug in traditional Chinese medicine to treat uterine prolapse, rectal prolapse, diarrhea, and other illnesses (Tang & Chen, 2005). Additionally, the fresh stems of *Ficus esquiroliana* contain a number of viscous white liquids. Therefore, on the basis of the above two characteristics, this plant might affect gastrointestinal motility and exacerbate the occurrence of ruminal impaction in Boer goats.

Ruminal impaction is considered an important internal disease in ruminants, such as dairy cows, sheep, and goats. It has been reported that it could be caused by many types of agents (Behera et al. 2013; Igbokwe et al. 2003; Jones & Money, 1965; Khose et al. 2010; Suthar et al. 2011). This study describes a novel case of ruminal impaction in Boer goats due to the plant *Ficus esquiroliana*. Breeders should pay particular attention to *Ficus esquiroliana* when providing food for ruminants.

## Conclusion

To our knowledge, it was the first time described that a novel case of ruminal impaction in Boer goats due to *Ficus esquiroliana*.
